# Attributes of host-specificity better explain the diversified wood-boring longhorn beetles in tropical SW China than plant species diversity

**DOI:** 10.1038/s41598-023-34511-2

**Published:** 2023-06-20

**Authors:** Fang Luo, Farkhanda Bibi, Terd Disayathanoowat, Tial C. Ling

**Affiliations:** 1 CAS Key Laboratory of Tropical Forest Ecology, Xishuangbanna Tropical Botanical Garden, Chinese Academy of Sciences, Mengla, 666303 Yunnan China; 2 Department of Botany, Garden Campus, Abdul Wali Khan University Mardan, Mardan, 23200 Pakistan; 3 Bee Protection Laboratory, Department of Biology, Faculty of Science, Chiang Mai University, Chiang Mai, 50200 Thailand

**Keywords:** Ecology, Plant sciences, Zoology

## Abstract

A long-debated question in ecology is whether the hyper-diversity of tropical plant-feeding insects is a direct consequence of high tropical plant diversity or should be attributed to increases in host plant specialization. In this study, we used Cerambycidae (the wood-boring longhorn beetles whose larval stages feed on the xylems of trees and lianas) and plants as study materials to explore which hypothesis is more favoured. Multiple analyses were used to show the differences in host specificity of Cerambycidae in tropical and subtropical forests. From these analyses, we found that the alpha diversity of beetles in tropical forests was significantly higher than that in subtropical forests but not in plants. The relationship between plants and beetles was also closer in tropical areas than in subtropical areas. Our results imply that the wood-boring longhorn beetles show higher degrees of niche conservatism and host-specificity in tropical forests than in subtropical forests. The high diversity of wood-boring longhorn beetles in tropical forests might be explained to a large extent by their more finely partitioned diet breadth.

## Introduction

The hyper-diversity of phytophagous insects in tropical areas is a common phenomenon, and its mechanism is still under debate. In explaining the hyper-diversity of Coleoptera, especially in the tropics^1^, the hypothesis that latitudinal gradients in biodiversity are related to niche breadth was proposed by Janzen^2^. According to Janzen’s hypothesis, the reduced seasonality in the tropics selects for narrower thermal tolerance in species, limiting the dispersal capacities along the tropical altitudinal gradients, such as warmer valleys to cooler mountain tops. These locally adapted traits would evolve into limited niche breadth and species specialization, promoting higher speciation rates in tropical mountains than in temperate ones^2^. An alternative idea is that tropical plant-feeding insects' species richness is a direct consequence of high tropical plant diversity rather than increased host plant specialization^3^. However, many studies focused on the diversity of different taxonomic or functional groups and testing one or the other above hypotheses have achieved contrasting conclusions^4–7^.

If the latitude-niche breadth hypothesis is preferred, there should be apparent patterns of specialization degree along the latitudinal gradients, which means tropical species would own narrower niche breadth, which equates to greater specialization. The beta-diversity of plant-phytophagous insect food webs has indicated the interaction preferences of insects with their host plants^8^. High beta-diversity values generally represent species compositions that differ remarkably in their community assemblages across time and space^9,10^. Generally, species at higher trophic levels depend more on species at lower trophic levels than vice versa. This dependence stems from the presence of at least one resource species as a prerequisite for the presence of the consumer species^11,12^. Thus, we can speculate that the beta-diversity variation of the consumers results from the species compositional change of the producers, as they create biotic filters for consumers that have close associations with them. Likewise, the dissimilarity in the composition of the producer species would scale up through the trophic chain and bring about the symmetrical pattern in the specialized consumers^13,14^. Nevertheless, the same is not necessarily valid for generalists. As they have a wider diet breadth, their host species can be freely distributed and exert no influence on their distribution^15^. Furthermore, generalists own a broader niche breadth compared to specialists. The spatial variation of species composition (beta-diversity) of generalists is commonly believed to reflect the extent to which they satisfy their niche requirements, and therefore, generalists get a lower value of beta-diversity compared with specialists^16^.

Although their matching patterns could indicate host specialization of phytophagous insects to their resource plants, it could also indicate parallel responses of both taxa to broad abiotic factors (macro-climatic gradients or shared historical processes). Therefore, patterns resulting from parallel responses to macro-climatic gradients or biogeographic histories are often difficult to distinguish from those resulting from biotic plant–insect interactions^17^. A potential approach to teasing these forces apart is to examine the patterns of association between plant and insect beta diversity at different spatial scales^18^. Usually, biotic interactions exert their influence at relatively small spatial scales. For example, some research pointed out that the effect of biotic interactions can be measured principally at the local scale^19,20^. In contrast, abiotic factors usually influence broader spatial scales^21,22^. Thus, we hypothesize that if both patterns result from insect-host specialization, plant and insect beta-diversity should be correlated at fine spatial scales. Alternatively, if the patterns result from parallel responses to broad abiotic gradients, the beta-diversity patterns should only be correlated at broad spatial scales^18^.

Longhorn beetles (Cerambycidae) belong to the order Coleoptera in the class Insecta. Their larvae usually burrow into the tissues (alive, moribund, dead, and decomposing) of woody plants^23^. Most longhorn beetles spend most of their life cycle within their host plant, in either the phloem or xylem of woody plants. It is estimated that there are more than 35,000 species of longhorn beetle worldwide, included in about 4000 genera^24^. The relationships between longhorn beetles and their host plants are often quite specific, while the breadth of host plant species used by the larvae of different species ranges substantially^25^. Our study uses longhorn beetle and plants as a study system to address the following questions relating to the hypotheses listed above: (1) Is there any relationship between the alpha-diversity and beta-diversity of plants and longhorn beetles in tropical forests and subtropical forests? (2) Is there a difference in host-specificity between longhorn beetles in tropical and subtropical forests at the community level? (3) Which mechanism, the higher degrees of host-specificity or the more diversified plant species, better explains the diversity of phytophagous insects in the tropics?

## Results

### Plant and insect composition and species diversity

We collected 625 individual longhorn beetles from tropical forests and classified them into 151 morphological species (Supplementary Material 1). Together, the five most abundant longhorn beetle morphological species accounted for 28.2% of the individuals collected (Fig. S3). A total of 917 individual trees from 186 species were recorded in the tropical forests (see Supplementary Material 2). The five most abundant plant species (with more than five individuals each) accounted for 26.4% of the individuals collected.

We collected 841 individual longhorn beetles from subtropical forests and classified them into 113 morphological species (see Supplementary Material 1). The five most abundant longhorn beetle species accounted for 48.3% of the individuals collected (Fig. S3). A total of 834 individual plants from 155 species were recorded in the subtropical forests (Supplementary Material 2). The five most abundant plant species (with more than five individuals each) accounted for 15.1% of the individuals collected.

The NMDS results offer a test of similarity between all plots. The results revealed that the transects generated clear clusters separating tropical and subtropical sites with well over-dispersed Ellipses (indicate 95% confidence intervals around centroids) in both plant (stress = 0.05793) and beetle (stress = 0.08923) communities after 20 iterations (no singletons and doubletons were excluded prior to analysis). These results indicate that the tropical and subtropical forest species were well stratified; thus, there were almost no shared species of both plants and beetles (Fig. S2).

**Figure 1:**
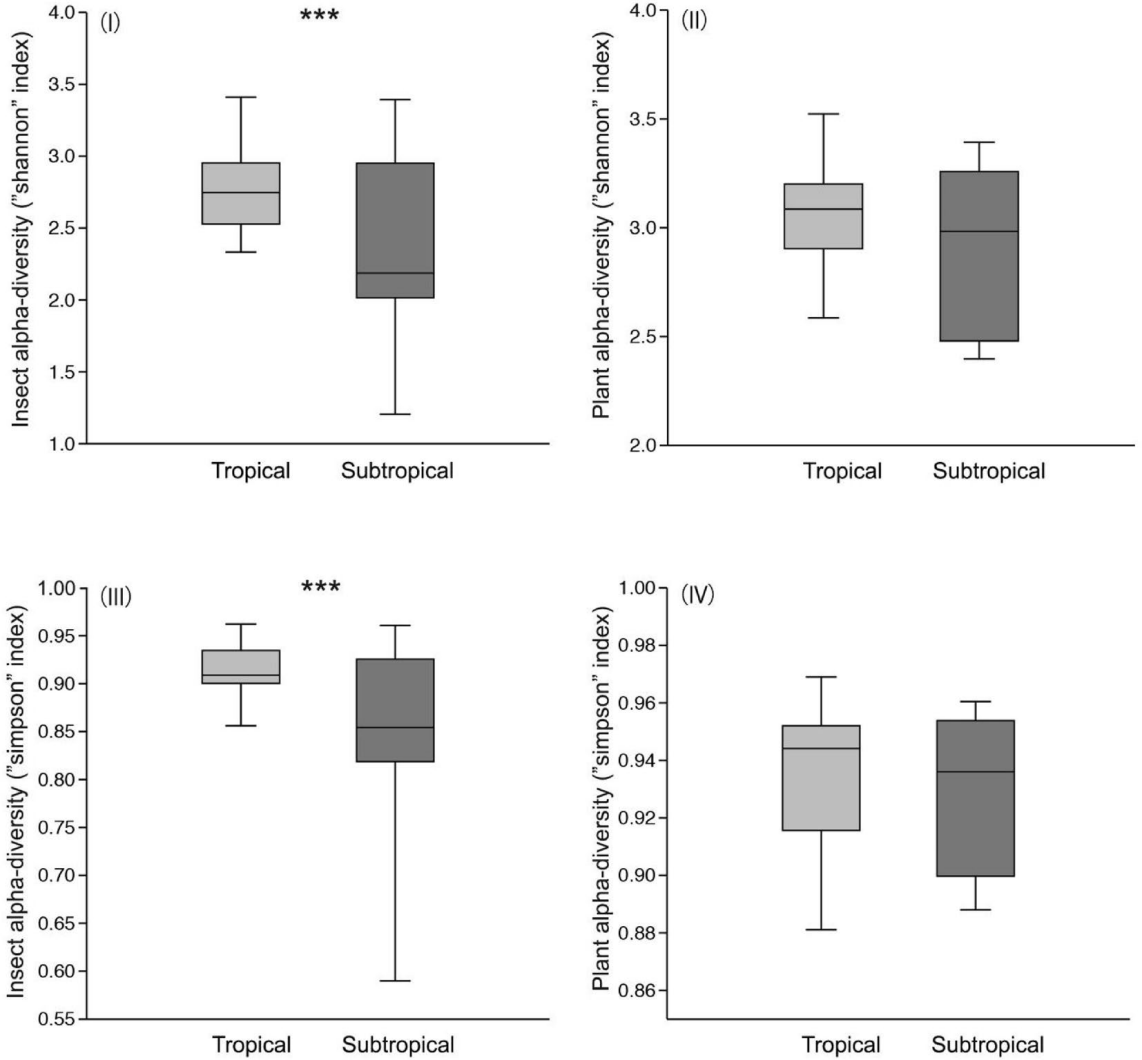
(**I**) shows estimated values of Shannon alpha-diversity of beetles in the tropics and subtropics, (**II**) shows estimated values of Shannon alpha-diversity of plants in the tropics and subtropics (**III**) shows estimated values of Simpson alpha-diversity of Beetles in the tropics and subtropics. (**IV**) shows estimated values of Simpson alpha-diversity of Plants in the tropics and subtropics. The box plots show the medians (horizontal line in box), 50% (the box limits), and 95% of all values (brackets). Wilcoxon tests were conducted to test for differences in alpha-diversity between the tropics and subtropics. ***indicates significant differences in the alpha-diversity of beetles or plants between the tropics and subtropics at *P* ≤ 0.05.

The Wilcoxon tests showed that the longhorn beetles in the tropical forest had a significantly higher Simpson and Shannon alpha-diversity value than those in the subtropical forests (Z = 1.931, P = 0.05; Z = 2.442, P < 0.05), but there was no significant difference in plant alpha-diversity (Simpson and Shannon index) between tropical and subtropical forests we sampled (Z = 0.7951, P > 0.05; Z = 1.534, P > 0.05) (Fig. 1).

### Spatial scale of species beta-diversity

**Figure 2:**
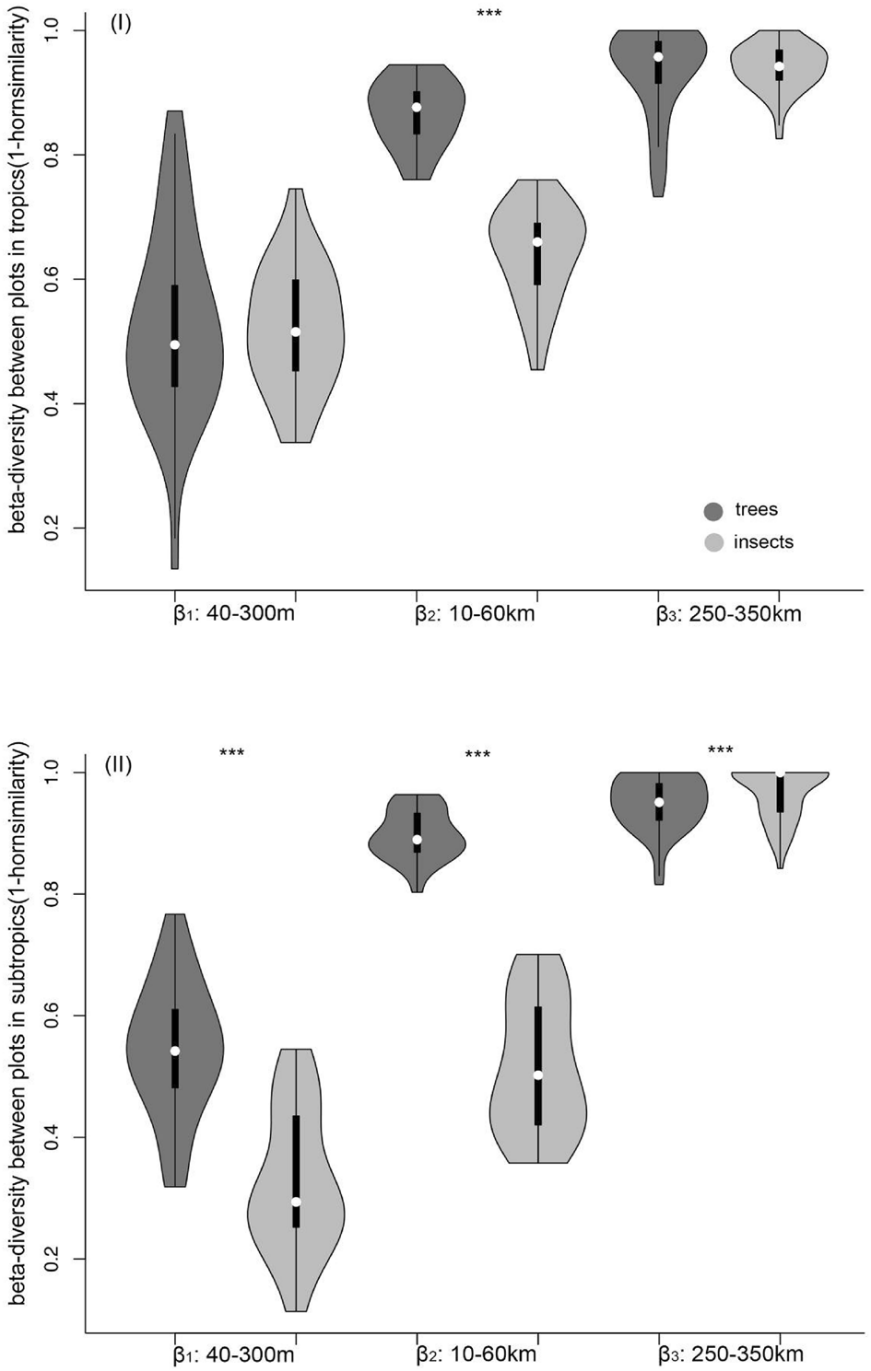
Beetle (dark grey) and plant (light grey) species beta-diversity (1–Horn similarity) between sampling plots (25 × 20 sq. m) with different spatial distances in tropical (**I**) and subtropical (**II**) areas in Yunnan province, SW China. The 1–Horn similarity value was calculated as the beta-diversity of (1) plots within a transect (β_1_: 40–100 m scale), (2) plots between two neighbouring transects within a region (β_2_: 10–60 km scale), (3) plots between two neighbouring regions (β_3_: 250–300 km scale). White dots represent medians, thick black bars represent first quartiles, and thin black lines represent the range. The shape of each plot shows the frequency distribution of the data. Wilcoxon tests were conducted to test for differences in beta-diversity values between beetles and plants at various spatial scales. ***indicates significant differences in beta-diversity between beetles and plants at each successive spatial scale at *P* < 0.05.

In the tropical area, the general pattern of plant species beta-diversity was similar to that of the beetles at the β_1_ (40–300 m) (Z = 0.134, P > 0.05, Fig. 2I)) and β_3_ (250–350 km) scales (Z = 0.025, P > 0.05, Fig. 2I), but at the β_2_ scale (10–60 km), the plant communities had a significantly higher beta-diversity value than the beetle communities (Z = 4.372, P < 0.001, Fig. 2I). Thus, plant and beetle compositional dissimilarity in the tropical area showed roughly similar trends with increasing spatial distances, with some differences at intermediate spatial scales.

In the subtropical area, the general pattern of plant species beta-diversity was significantly different from that of the beetles at the β_1_ (40–300 m) (Z = 4.782, P < 0.001, Fig. 2II) and β_2_ (10–60 km) scales (Z = 4.372, P < 0.001, Fig. 2II), but not at the β_3_ scale (250–350 km) (Z = 2.332, P > 0.05, Fig. 2II). Thus, plant and beetle compositional beta-dissimilarity showed significantly different spatial distances in the subtropical area, except at the macro-scale.

**Figure 3:**
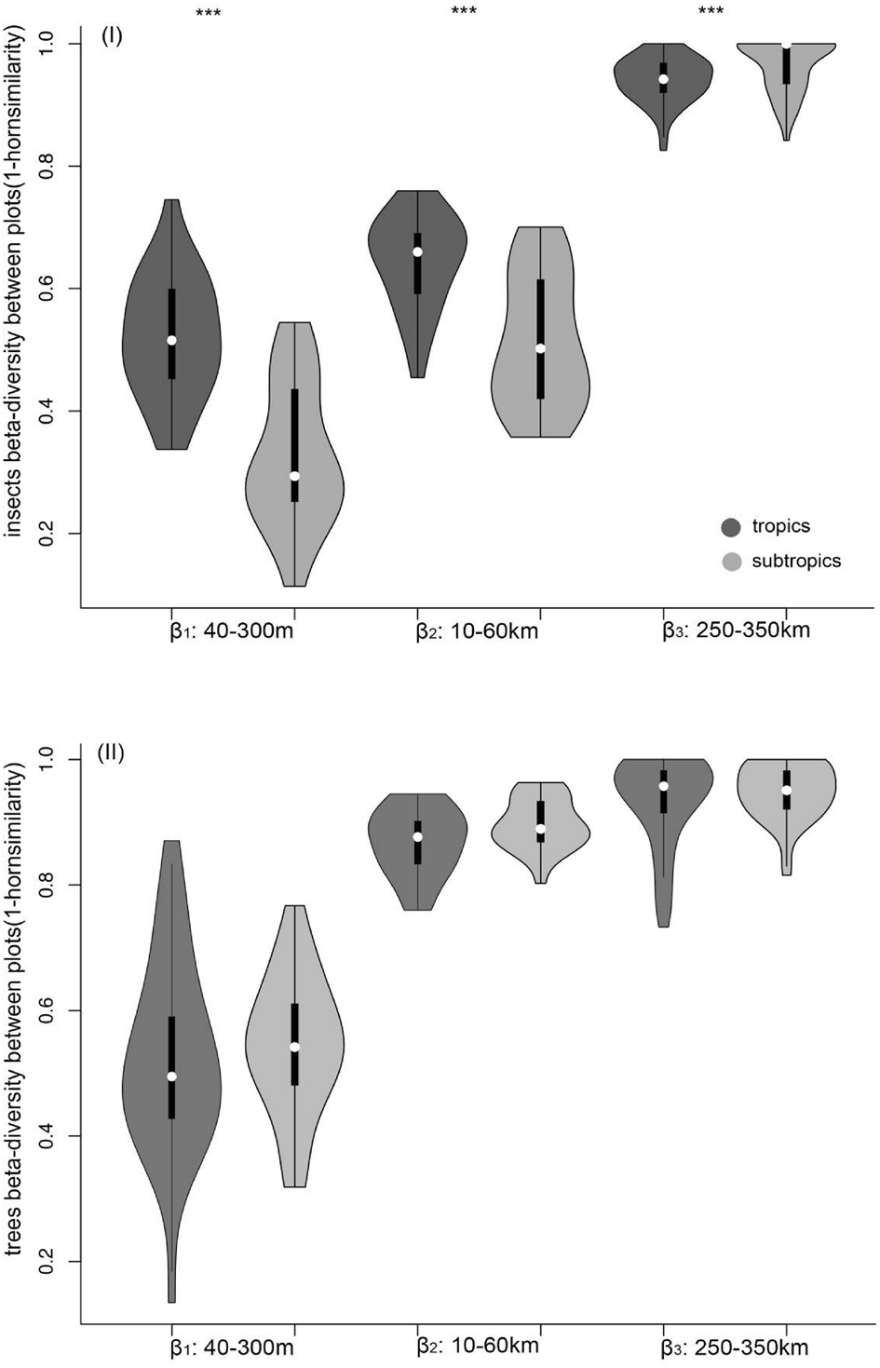
(**I**) represents beetle beta-diversity (1–Horn similarity) between sampling plots with different spatial distances in the tropics (dark grey) and subtropics (light grey) in Yunnan province, SW China. (**II**) represents plant beta-diversity (1–Horn similarity) between sampling plots with different spatial distances in the tropics (dark grey) and subtropics (light grey) in Yunnan province, SW China. The 1–Horn similarity value was calculated as the beta-diversity of (1) plots within a transect (β_1_: 40–300 m scale), (2) plots between two neighbouring transects within a region (β_2_: 10–60 km scale), (3) plots between two neighbouring regions (β_3_: 250–350 km scale). White dots represent medians, thick black bars represent first quartiles, and thin black lines represent the range. The shape of each plot shows the frequency distribution of the data. Wilcoxon tests were conducted to test for differences in beta-diversity values between beetles and plants in the tropical and subtropical region along various spatial scales. ***indicates significant differences in beta-diversity between beetles and plants at each successive spatial scale at *P* < 0.05.

The general patterns of beetle species beta-diversity between the studied tropical and subtropical areas were significantly different at the β_1_ (40–300 m), β_2_ (10–60 km), and β_3_ (250–350 km) scales. The Wilcoxon tests showed that the beetle communities in the tropical areas had significantly higher beta-diversity values than those in the subtropical areas at the β_1_ (Z = 4.188, P < 0.001, Fig. 3I) and β_2_ scales (Z = 3.458, P < 0.001, Fig. 3I), but significantly lower beta-diversity values at the β_3_ scale (Z = 3.005, P < 0.05, Fig. 3I). Therefore, the beetle communities generally showed higher beta-diversity values in the tropical areas than in the subtropical areas, except at the macro-scale.

**Figure 4:**
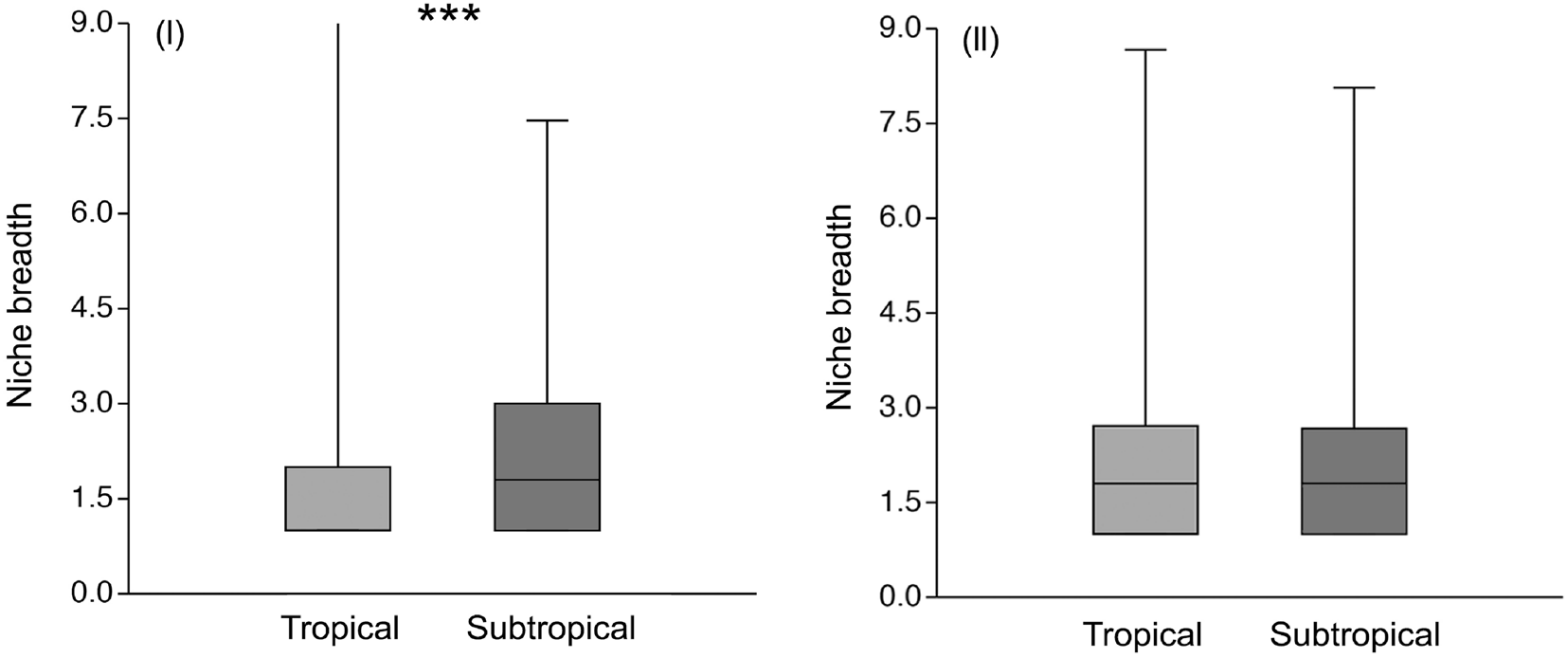
Community assembly measured as habitat niche breadth of beetles (**I**) and plants (**II**) in the tropical and subtropical forests. The box plots show the medians (horizontal line in box), 50% (the box limits), and 95% of all values (brackets). Kruskal–Wallis tests were conducted to test for differences in beetles and plant niche-breadth between the tropics and subtropics. ***indicates significant differences at *P* ≤ 0.05.

The general patterns of plant species beta-diversity between the tropical and subtropical areas were similar at the β_1_ (40–300 m) (Z = 0.545, P > 0.05, Fig. 3II), β_2_ (10–60 km) (Z = 1.359, P > 0.05, Fig. 3II), and β_3_ (250–350 km) scales (Z = 0.910, P > 0.05, Fig. 3II). Thus, plant compositional beta-dissimilarity in the tropical and subtropical areas showed roughly similar patterns with varying spatial distances. The niche breadths of tropical beetles were lower compared to subtropical beetle assemblages (P < 0.05, Fig. 4I), while the niche breadths of tropical and subtropical plants were not significantly different (P > 0.05, Fig. 4II).

### Correlates of insect compositional assemblage

**Figure 5:**
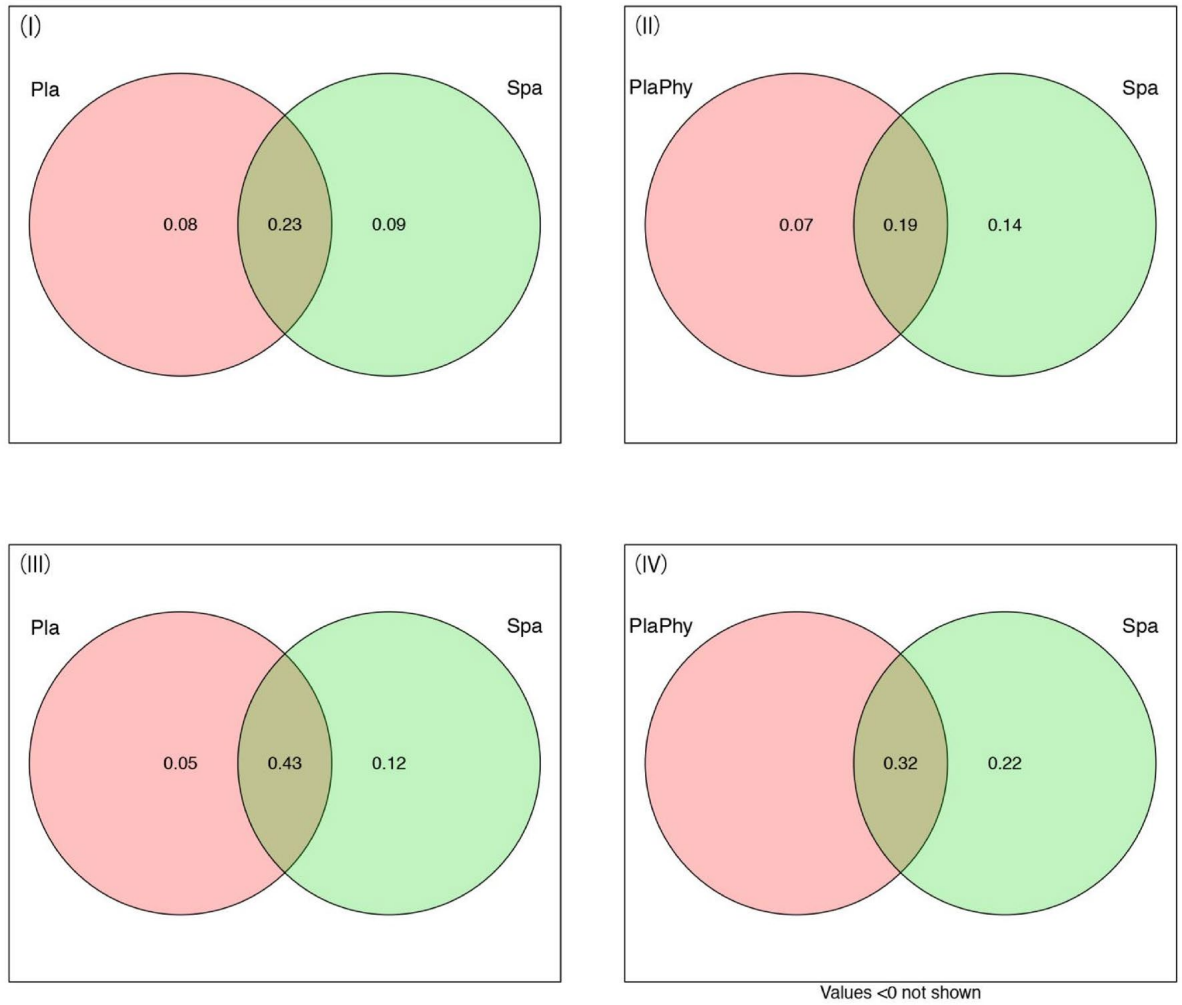
Variation partitioning analyses depicting both the isolated and combined explanatory power of distance with plant species and plant phylogenetic beta-diversity in wood-boring longhorn beetle composition in tropical and subtropical areas in Yunnan province, SW China. (**I**) and (**II**) represent the tropical areas, (**III**) and (**IV**) represent the subtropical areas. Pla:plant species, PlaPhy:plant phylogenetic beta-diversity, Spa:spatial distance. The numerical values within the circles indicate the explanatory power of the counterparts, value < 0 not shown.

In the tropical forests, RDA variation partitioning revealed that the effect of geographical distance, plant species diversity, and phylogeny separately explained 23% and 19% of the variation in beetle species composition at the macro-scale. The pure effects of plant species diversity and phylogeny were 8% (Fig. 5I) and 7% (Fig. 5II). In each case, the pure effects of geographic distance were 9% (Fig. 5I) and 14% (Fig. 5II).

In the subtropical forests, RDA variation partitioning revealed that the combined effect of geographical distance, plant species diversity, and plant phylogeny separately explained 43% and 32% of the variation in beetle species composition at the macro-scale. The pure effects of plant species and plant phylogeny were 5% (Fig. 5III) and < 0 (Fig. 5IV), respectively. At the same time, the pure effects of geographic distance in each case were 12% (Fig. 5III) and 22% (Fig. 5IV).

**Figure 6:**
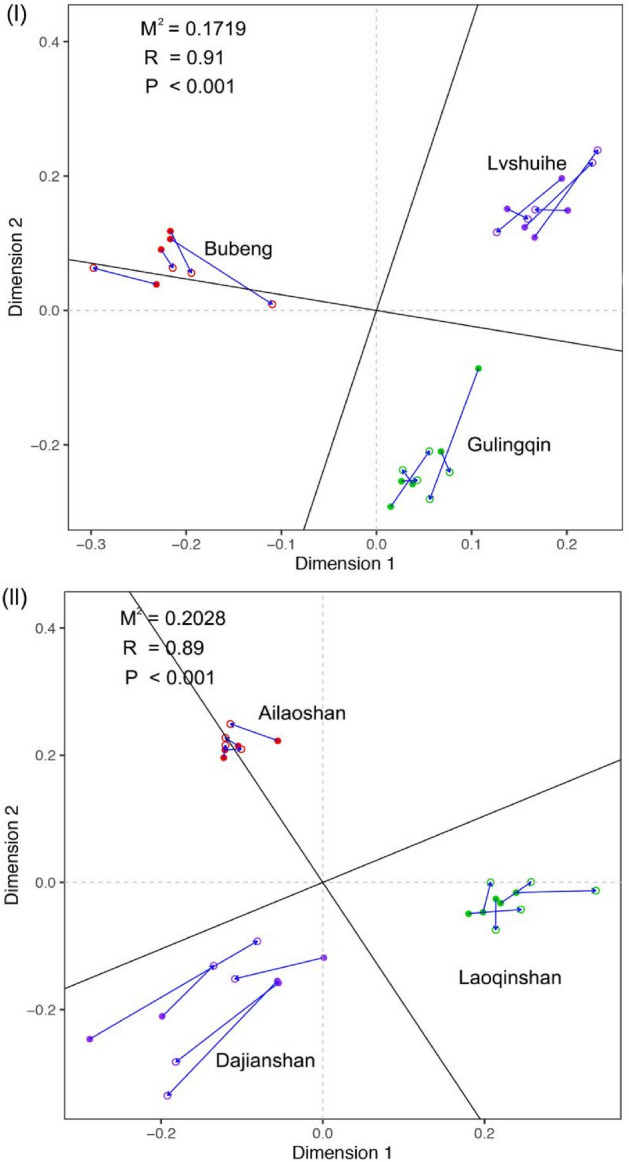
Procrustes analysis depicting the correlation between plant composition and wood-boring longhorn beetle composition in tropical (**I**) and subtropical (**II**) areas on the basis of Bray − Curtis distances (9999 permutations). M^2^ is a measure of fit provided, the smaller the M^2^, the closer the relationship between the plant community composition and beetles’community composition. P < 0.001 means the two data sets exhibit greater concordance than expected at random.

For the Procrustes analysis, we can see that in the tropical area, the plant communities explained about 91% variation of the insect communities, the M2 is 0.1719 (Fig. 6I), while in the subtropical area, the plant communities explained about 89% variation of the insect communities, the M2 is 0.2028 (Fig. 6II), the closer relationship between the two data sets is indicated by the smaller M2 value. And the two data sets exhibit greater concordance than expected at random, with P < 0.001 in both tropical and subtropical area.

## Discussion

Our results reveal that changes in the composition of insect herbivore communities track changes in plant species in tropical forests. First, the beta-diversity comparison showed that the beetle communities were strongly linked to plant communities across remarkably short spatial scales (i.e. β1: 40–300 m, Fig. 2I). At the higher trophic level, the beetle species are more dependent on plant species at lower trophic levels. If the beetle species are host-specialized, they will generate symmetrical patterns with plant species and reflect resource dissimilarity^12^. Meanwhile, this is a specific range of the scale at which biotic interactions play a role in community assembly^26^, pointing to host-specificity as the underlying process rather than shared bio-geographical histories or parallel responses to climatic gradients, as this fine spatial scale would not impose prominent environmental gradients to insect community assemblage, the potential driving forces of community assembly is largely explained by the biotic interaction^18,19,27^. Second, the variation partitioning analysis results show that, alone, plant species composition and phylogeny accounted for 8% and 7% of the explained variation in wood-boring longhorn beetle community composition in tropical forests. This means that after removing the effect of geographic distance, the beetles and plants are still positively correlated, indicating that the host-specificity of Cerambycidae is the potential mechanism contributing to the presented pattern in tropical forests. From the Procrustes analysis, we can see that in the tropical area, the plant communities explained about 91% variation of the insect communities, and a smaller M^2^ = 0.1719 compared to the subtropical area (M^2^ = 0.2028), P < 0.001 indicate that the two data sets showed greater concordance than expected at random.

However, the same statistical methods produced different results in the subtropical forests. This study shows weak evidence that changes in the composition of the beetle communities follow plant community composition and plant phylogeny in subtropical forests. The beta-diversity comparison results showed no indication that beetle and plant compositional variation was reflected at changing spatial scales. Also, the variation partitioning results show that after removing the influence of geographical separation, plant species composition and phylogeny separately explained 5% and < 0 of the variation in beetle community composition.

A different perspective drawing the same conclusion is illustrated from niche breadth analysis and separate beta-diversity comparison of insects and plants. From niche breadth analysis, we can see those tropical beetles obtain lower niche breadth than subtropical beetle assemblages. In contrast, the niche breadths of tropical and subtropical plants were not significantly different. The plant beta-diversity comparison showed that the plant turnover in the tropics mirrored that in the subtropics at multiple spatial scales; however, for the beetle communities, beta-diversity in the tropical area was significantly higher than that in the subtropical area at almost all spatial scales. This divergence indicates that the longhorn beetles, as plant-feeding insects, hold higher degrees of niche conservatism in the tropics than in the subtropics. We can speculate that this pattern also indicates higher degrees of host-specificity of insects in the tropical zone than the subtropical ones.

In this study, plant alpha-diversity showed no significant difference between the tropics and subtropics, but beetle alpha-diversity was significantly higher in the tropics than in the subtropics. Therefore, our study did not strongly support the hypothesis of Novotny et al.^8^ that the hyper-diversity of plant-feeding insects in tropical areas is the direct result of the greater diversity of plants. However, Janzen’s hypothesis, which explains that higher degrees of host-specificity are the driving forces in the diversification of insects in the tropics, tends to be supported^28–30^. Longhorn beetles, as host specialists, would inhabit narrower niche widths in the tropics relative to subtropics compared with plants. Therefore, plants and insects gain higher diversity indices in the tropics, but only insects are significantly higher. Another explanation for this phenomenon is that hyper-diverse groups like trees in tropical rainforests are characterized more by functional redundancy than niche specialization. For example, more than 80% of neotropical trees are shade tolerant, and these shade-tolerant species do not appear to require a finely partitioned abiotic niche^31^.

Janzen’s hypothesis bridges the traditional contradictory two perspectives (i.e. evolution vs. ecology) in explaining species diversity along the climatic gradients. It combines climate patterns and adaptation mechanisms to explain macro-ecological phenomena by depicting climatic variability for shaping the evolution of species’ physiological traits and geographical range sizes^2,32^. This pattern is not limited to longhorn beetles, and many other studies have documented significant differences in species richness between climatic gradients and different habitats related to niche-breadth^4–6^.

## Conclusions

Our study demonstrates that the host plant specialization in wood-boring longhorn beetles changes with the climatic zone. Furthermore, it implies that greater ecological specificity contributes to higher alpha- and beta-diversity in plant-feeding insects, highlighting the key role of ecological specialization in the coexistence and diversification of insect species. This partly explains why there are so many species of phytophagous insects in the tropics. We cannot deny the fact that plant diversity has a profound impact on the structure of plant-feeding insect communities and maintaining biodiversity. However, compared with host-specificity and niche conservatism, the importance of plant species diversity might not be that pronounced. Our study demonstrates how species host-specificity might shape wood-boring longhorn beetle diversity patterns through different climatic zones.

### Limitation of study and recommendations

Although our current study exhibits a trend toward a high diversity of wood-boring longhorn beetles in tropical forests when compared to subtropical forests, we cannot confirm that such changes in host plant specialization and plant-feeding insect communities were solely due to the variation of the diversity of plant species across altitudinal gradients. Significant changes in the structure of plant community and diversity, such that tropical forests compose plants with more complex canopy strata compared to subtropical and temperate forests^33^, coupled with environmental mechanisms, might be significantly responsible for the diversity and feeding behaviours of longhorn beetles. Unfortunately, the present study did not measure such important parameters. Therefore, further investigation is required to validate the significant importance of beetles that feed on understory plants to avoid sampling bias. Such sampling methods include the use of pheromones to attract more beetles and the use of mix traps of various colors, instead of transparent traps which can collect more specimens^33,34^; and further classify the Cerambycidae into specialists or generalists. Nevertheless, we recommend that more in-depth studies on biotic interaction-induced niche conservatism are urgently needed if we are to attain a better understanding of the origin, maintenance, functioning, and management of tropical biodiversity, and the exploration of ecological and evolutionary underpinnings of niche-breadth will allow us to generate more robust predictions, range dynamics, evolutionary trajectories, and the maintenance of biodiversity and ecosystem functions.

## Methods

### Study sites and study system

This study was conducted at the following six sites, located in tropical and subtropical zones, each one of them described below:

#### Site 1

**Figure 7:**
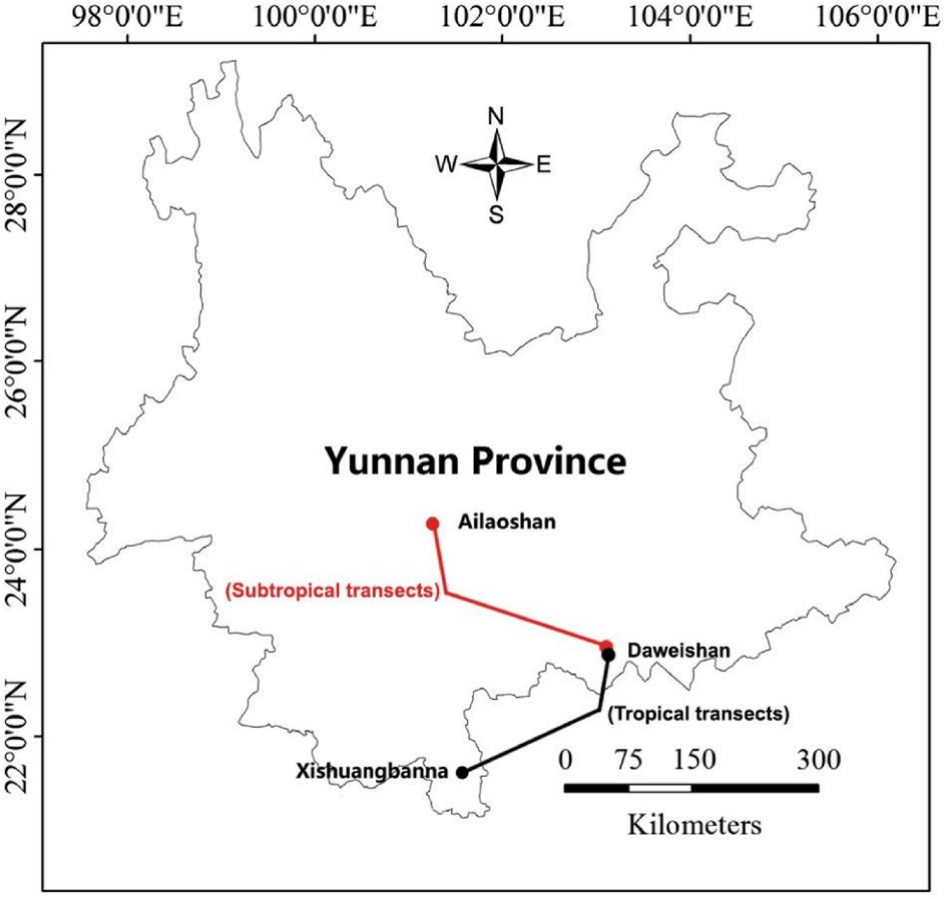
Geographical locations of the six study sites in three regions within two different climatic zones of Yunnan province, Southwest China. The red points represent subtropical transects, and the black points represent tropical transects.

Bubeng, Xishuangbanna (21.61°N, 101.58°S) (Fig. 7), is a tropical rainforest transect with an elevation of about 600 m, located within the Xishuangbanna National Natural Reserve. The climate in this area is monsoonal, with a pronounced wet season between May and October and a dry season between November and April. Mean annual temperature and rainfall are 22 °C and 1500 mm, respectively.

#### Site 2

Xujiaba, Ailao Mountains (24.53°N, 101.03°S), is located within the large Ailao Mountain National Natural Reserve approximately 329 km from Bubeng. It is a high-elevation subtropical transect with an elevation of about 2200 m (Fig. 7). The mean annual temperature and rainfall are 11 °C and 1900 mm, respectively, with a dry season between December and April. The Ailao Mountains occur at a significant climatic boundary between the southwest and southeast monsoon systems of China.

#### Sites 3–6

Dawei Mountains (22.50°N, 103.40°S) (Fig. 7) are located within the Dawei Mountains Reserve. Dawei Mountain range is a boundary between tropical and subtropical areas where two sites are considered tropical, and the other two are subtropical. Two tropical sites were about 68 km apart, with elevations of about 600 m (Gulingqin and Lvshuihe). The remaining two were in the subtropical area, about 10 km apart, with elevations of about 2200 m (Dajianshan and Laoqinshan). The distance between the tropical and subtropical sites was about 40 km. The mean annual temperature and rainfall in the lowland tropical areas (Gulingqin and Lvshuihe) are 22 °C and 1764 mm, respectively, with a dry season between November and April, a typical tropical monsoon climate. The climate at Gulingqin and Lvshuihe essentially differs from that of Dajianshan and Laoqinshan, owing to their lower altitude. Dajianshan and Laoqinshan have almost the same altitude as the sampling site in the Ailao Mountains (site 2). These three sites were all regarded as subtropical.

### Insect and plant sampling

Five 25 × 20 m plots (covering 0.25 ha) were set up at each site, spaced at least 40 m apart. The area sampled at each site followed the recommendations of Zhu et al.^35^. All the plots were established away from large canopy gaps created by recent anthropogenic or natural disturbances. Beetle sampling was conducted using one modified aerial collector in the canopy areas of each plot at each site. Usually, the standard way is to sample insects in the canopy and understory. However, we obtained very few species in the understory in our pre-experiment, and therefore we sampled the species only in the canopy. Unmanned aerial vehicles and pulling ropes were used to install flight intercept traps (FITs) in the canopy. The FITs were constructed from two rigid, transparent plastic plates (50 × 35 cm, height × width), which were arranged crosswise and fixed above a red or blue plastic bowl (35 × 30 cm, diameter × height). A round, transparent, soft plastic plate with a diameter of 45 cm roofed each FIT to minimize entering rainwater during the rainy season. Within each plot, one trap was installed in the canopy hanging on a branch at the height of 15–20 m above the ground, depending on the canopy height of each plot. The collecting basins of the FITs were filled with a liquid mixture of 75% alcohol and blue anti-freeze (ethanol-glycol) at 1:2 v/v^36^. The number of red and blue bowls was almost equally distributed in each site to reduce the potential bias^34^.

We matched our insect sampling with longhorn beetle abundance peaks from the beginning of March 2018 to the end of June 2018. The traps were emptied every ten days during the collection period. The specimens caught in the alcohol and anti-freeze mixture were filtered and preserved in 70% ethanol. After initial specimen cleaning (removing the trash and untargeted insects), the morphological classification was done by taxonomists experienced in Cerambycidae identification based on the Cerambycidae taxonomic books^37–39^. The abundance and richness of longhorn beetles in each trap were recorded. Voucher specimens of the collected beetles were stored temporarily in a laboratory at Honghe University and subsequently transferred to the National Zoological Museum of China, Institute of Zoology, Chinese Academy of Sciences, Beijing.

Vegetation data were collected in April, May, and October 2019. We surveyed the woody plants in each plot by measuring the abundance of each plant species (or morpho-species) ≥ 5.0 cm diameter at breast height (1.2 m). All sampling methods used in the present study comply with the Center for Tropical Forest Science (http://www.ctfs.si.edu/) instructions for the assembly of long-term, large-scale forest data from the tropics^40^ and with the Chinese Forest Biodiversity Monitoring Network (http://www.cfbiodiv.org/). Voucher specimens were collected whenever necessary in the field for later identification with the help of plant taxonomists.

### Data analysis

To clarify whether the beetle and plant species in the tropical and subtropical areas showed significantly different patterns, we calculated their Simpson and Shannon alpha-diversity. We used Wilcoxon tests to identify significant differences between the two climatic zones. Additionally, we also used a non-metric multidimensional scaling (NMDS) method to test for areas of high endemism by searching for readily apparent stratified species compositions in the different climatic zones. The presence of strongly delineated circles in the NMDS would indicate that the species composition of the plants and beetles in both the tropics and the subtropics is highly distinguished. The analysis was based on the Bray–Curtis dissimilarity distances of presence-absence data, performed with the ‘metaNMDS’ function in the package ‘vegan’^41^.

To clarify whether the wood-boring longhorn beetles showed host-specificity, we compared the beta-diversity values of the beetle and plant communities in two different climatic zones (tropics and subtropics) along with increasing spatial scales from local to regional (40 − 300 m, 10 − 60 km, and 250 − 350 km). We hypothesized that if beetle compositional variation tracked that of the plant community at a local scale, beetle-host specificity could contribute to beetle diversity. Furthermore, if the beetle and plant community beta-diversity patterns within different climatic zones (tropics and subtropics) are compared at increasing spatial scales (from local to regional scale), and a difference is found in the niche-breadth of the beetles between the two climatic zones, a differentiated beetle beta-diversity value will be produced. We also examined whether the plant species composition and phylogenetic beta diversity are good predictors of beetle community composition.

To calculate the alpha-diversity and beta-diversity of the beetles, we used data from the canopy FITs in each plot combined as the smallest sampling unit for diversity estimation. Plant diversity was recorded per plot. Alpha-diversity describes the species diversity in an individual plot. It is measured either by the number of species present in the plot (species richness) or by other functions (diversity indices) that consider the species' relative frequencies. In this study, we choose the most widely used of these indices: the Simpson^42^ and Shannon^43^ index. Beta-diversity was calculated using the package 'vegetarian'^44^ in R^45^ with the Horn similarity index. We visualized the beta-diversity as 1 − Horn similarity (i.e. compositional dissimilarity), such that values of 1 indicated complete species turnover between sampling units. Additionally, geographic distance matrices were constructed at the plot and transect levels using the function ‘earth.dist’ in the R package ‘fossil’^46^. As we were computing and comparing the species between identical sampling units in all cases, the species accumulation curve was not that important, but it tended toward saturation which indicated that the samples were sufficient to reveal the plant and insect communities (Fig. S1).

For the plant phylogenetic beta-diversity, all the enumerated species' family and genus names (215 species in total) were obtained with the R package ‘plantlist’^47^. Then, their phylogenetic relationships were examined using the online Phylomatic tool^48^ (www.phylodiversity.net/phylomatic/) based on the Angiosperm consensus of Davies et al.^49^. Further, similarity matrices were constructed for the plant phylogenetic beta-diversity, PhyloSor Index, Bryant et al.^50^ with the function ‘phylosor’ in the ‘picante’ package in R^51^. PhyloSor is a modified Sørensen similarity index that quantifies communities' phylogenetic similarity as the proportion of shared phylogenetic branch-lengths between two samples.

To examine longhorn beetle specialization in tropical and subtropical forests, we separately compared the beetle and plant beta-diversity results in the tropical and subtropical forests, at various spatial distances. We calculated the beetle and plant species dissimilarity of (1) plots within a transect (β1: 40 − 300 m scale), (2) plots in two neighbouring transects within a region (β2: 10 − 60 km scale), and (3) plots in two neighbouring regions (β3: 250 − 350 km scale). The spatial coordinates were converted into Cartesian coordinates. For the subtropical forests, the same procedures were followed. Wilcoxon tests were used to assess whether beta-diversity differed significantly between plants and beetles at each respective separation distance (i.e. β1, β2, β3).

To examine whether longhorn beetle specialization level differs between tropical and subtropical forests, we first compared the tropical and subtropical plant beta-diversity at different spatial scales (i.e. β1, β2, β3), we then compared the tropical and subtropical beetle beta-diversity at different spatial scales (i.e. β1, β2, β3). Wilcoxon tests were used to assess whether species variation differed significantly for plants and beetles in the tropics and subtropics at each respective separation distance (i.e. β1, β2, β3). P-values were adjusted for multiple comparisons.

To determine whether plant species composition and phylogenetic composition are good predictors of beetle species assemblage, we developed an ordination (redundancy analysis, RDA) approach to test the explanatory power of plant species and phylogeny, combined with spatial distance, in the beetle composition^52^. Principal component analysis (PCA) for plant species was performed using the ‘prcomp’ function in the ‘stats’ package, while the PCA for plant Phylogeny was conducted using ‘phyl.pca’ function in ‘phytools’ package (Table S1). We used backwards selection (‘ordistep’ function in the ‘vegan’ package) to assess the influence of plant species and plant phylogeny on beetle composition. We further used variation partitioning analysis through RDA to assess both unique and shared percentage contributions of each group of predictor variables (i.e. plant species composition/plant phylogeny and spatial distance) to explain the variation in abundance-based longhorn beetle composition. Variation partitioning analyses were performed in R using the ‘vegan’ and ‘packfor’ packages. The significance of the testable fractions (P ≤ 0.05) was based on 999 permutations. To further confirm the relationship between plant and beetle community structures along the six different sampling strata, we used Procrustes analysis depicting the correlation between plant composition and wood-boring longhorn beetle composition in tropical and subtropical areas on the basis of Bray − Curtis distances^53^.

The niche breadth of beetles in tropical and subtropical forests was respectively calculated with function 'niche.width’ in R package ‘spaa’, according to Levin’s niche breadth index^54^. Kruskal–Wallis tests were conducted to test for differences in beetles and plant niche-breadth between the tropics and subtropics. ***indicates significant differences at P ≤ 0.05. All analyses were performed in R 3.4.5^45^.

### Animal research (Ethics)

The institutional Animal Care approved all the animal samplings described in this work and Use Committees at Xishuangbanna Tropical Botanical Garden, and the Nature Reserve authorities of Yunnan, China.

## Supplementary Information

Supplementary Information 1.

Supplementary Information 2.

Supplementary Information 3.

## Data Availability

The datasets generated and/or analysed during the current study are available in the Dryad repository [https://doi.org/10.5061/dryad.6m905qg47]. Supplementary Material 1 contains the Cerambycidae species collected in different sampling plots. Supplementary Material 2 contains the plant species collected in different sampling plot.
