# Supplementary material for: Attributes of host-specificity better explain the diversified wood-boring longhorn beetles in tropical SW China than plant species diversity
Source: Sci Rep. 2023 Jun 20;13:9997. doi: 10.1038/s41598-023-34511-2 (PMC10281982; doi:10.1038/s41598-023-34511-2)
Supplement: Supplementary file 3 — Supplementary Information 3. [file 41598_2023_34511_MOESM3_ESM.docx]

**Supplementary Figures and tables**

**Figure S1.** Species accumulation curves of plants based on the 15 plots in the tropical and subtropical areas separately, Figure S2-(I) and Figure S2-(III) represents the tropical area, Figure S2-(II) and Figure S3-(IV)represents the subtropical area.

.


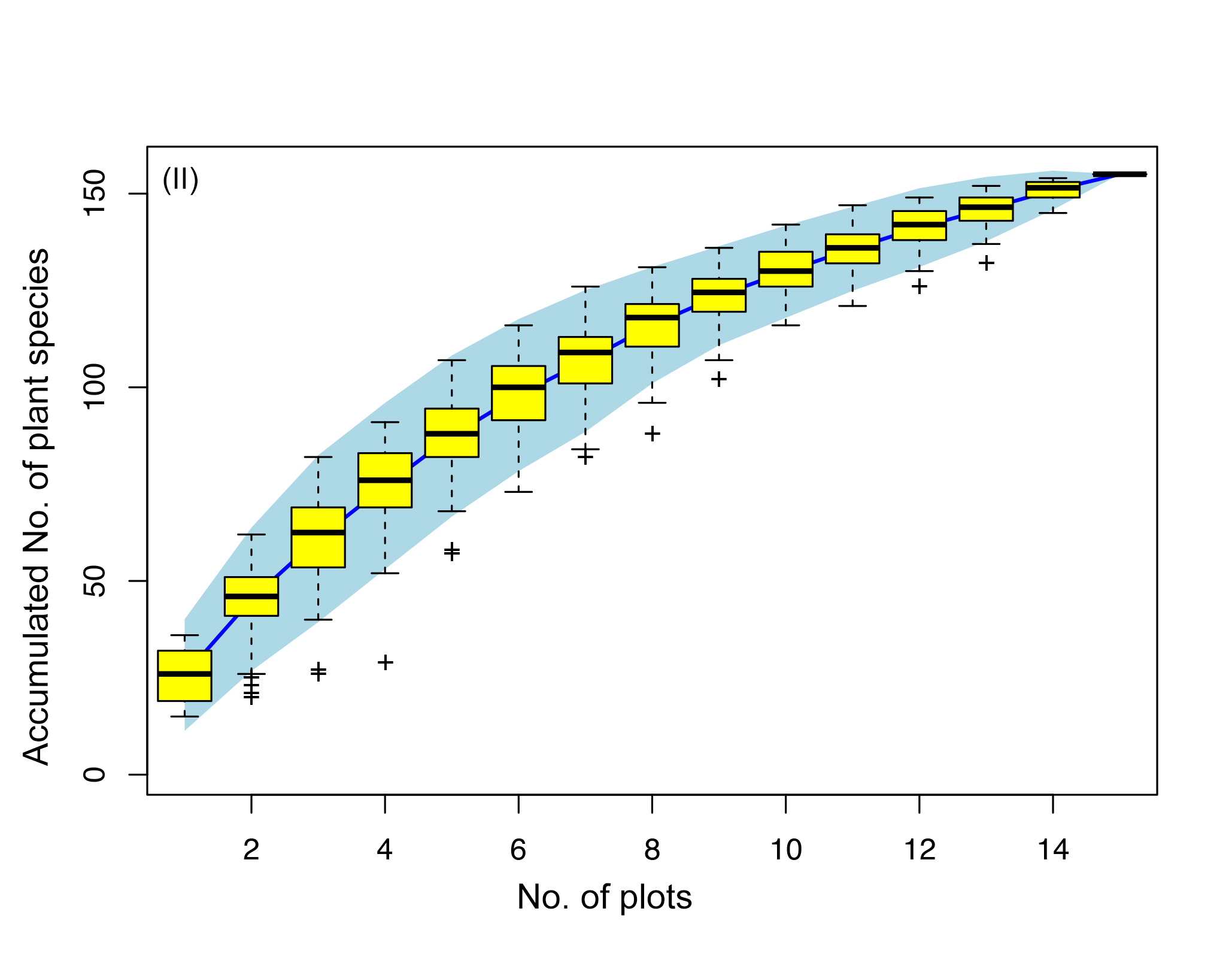

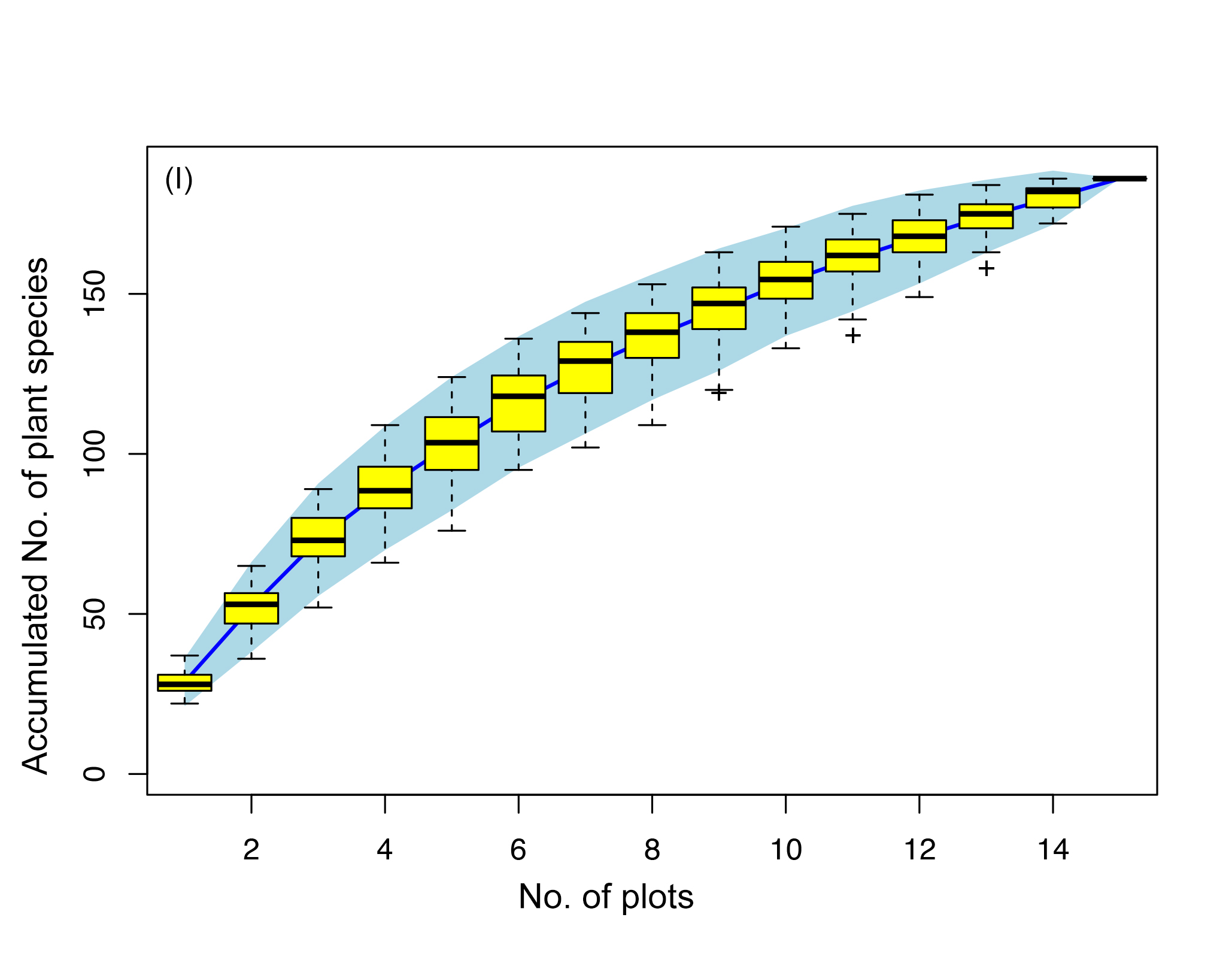


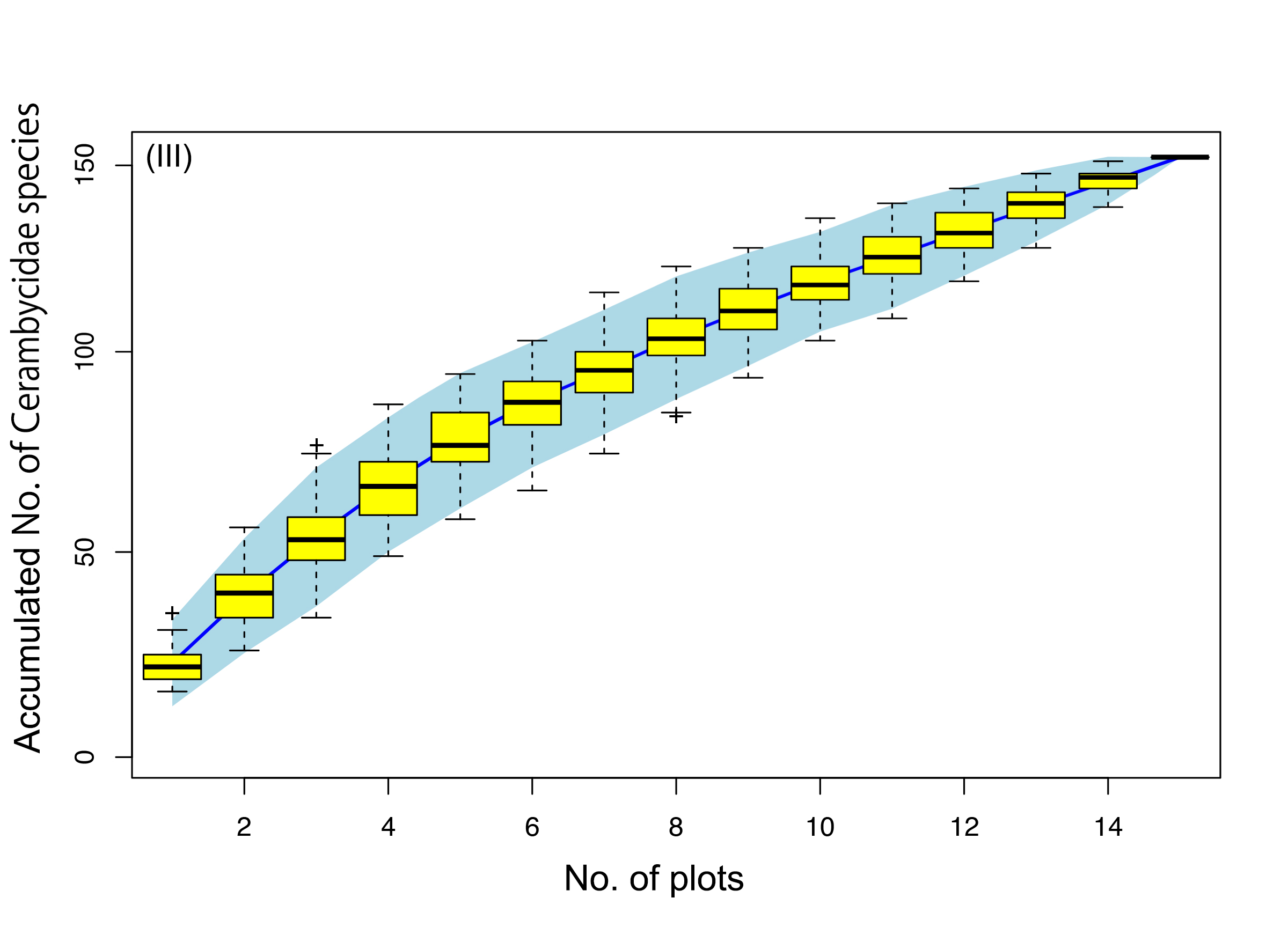


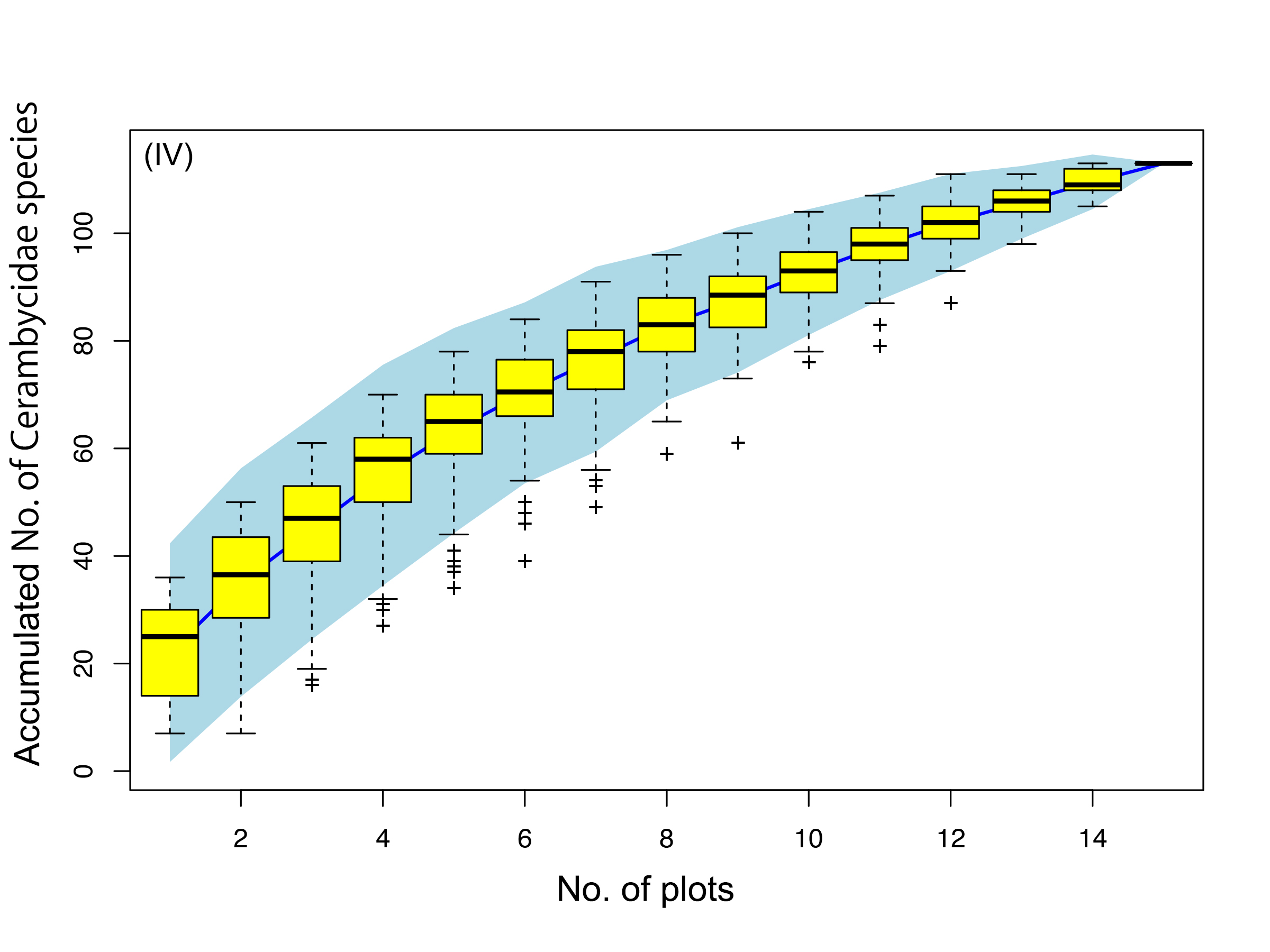


**Figure S2.** Non-metric multidimensional scaling (NMDS) ordinations for beetle assemblages (Fig. 2-I) and plant assemblages (Fig. 2-II) in different sampling plots. The red points are sampling plots, different numbers in the squares represent different transects (1: Ailaoshan; 2: Dajianshan; 3: Laoqinshan; 4: Bubeng; 5: Lvshuihe; 6: Gulingqin), the coloured ellipses are 95% confidence intervals of species centroids for each treatment level. Two convergent solutions were found after 20 iterations with a stress value of 0.05793 for plants and 0.08923 for beetles using Bray-Curtis distance.


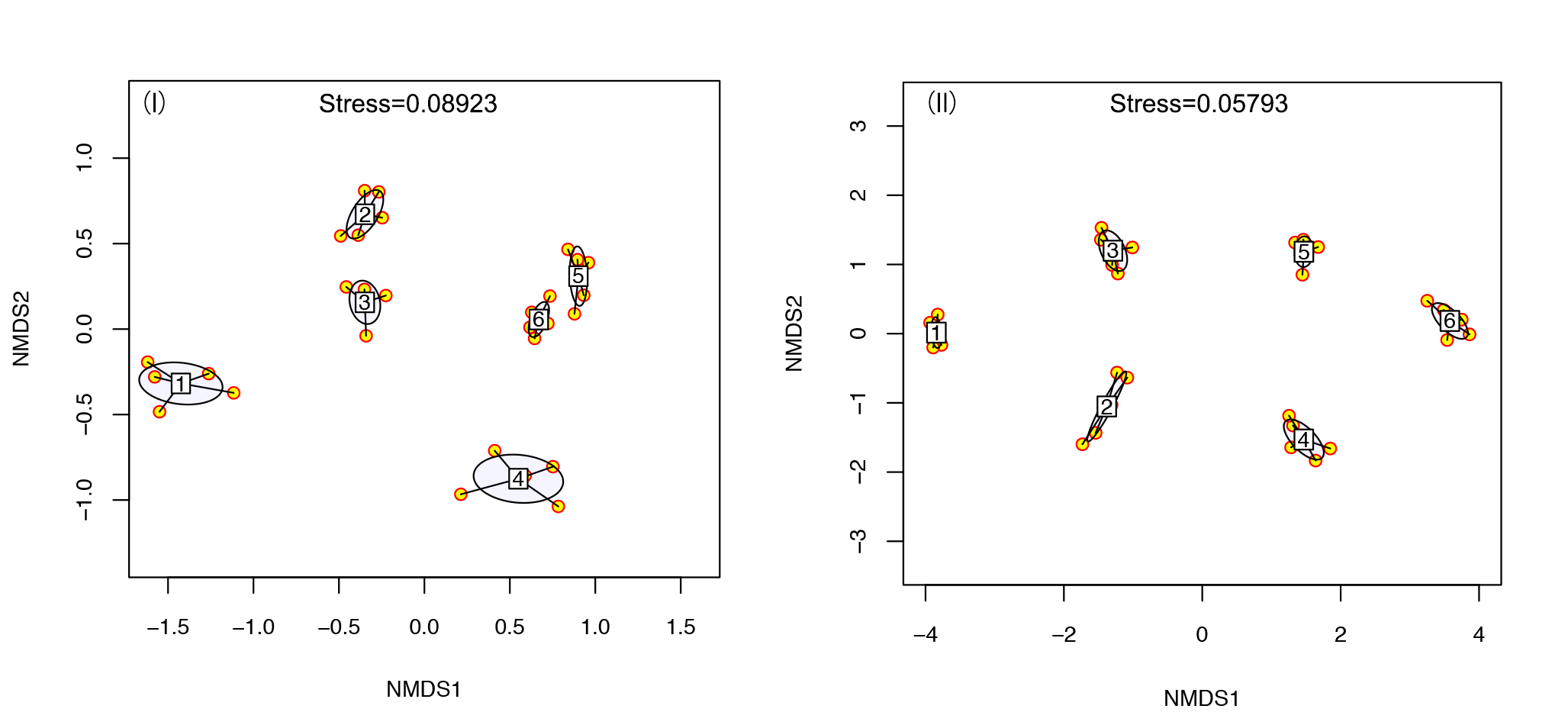


**Figure S3.** The proportion of the five most abundant longhorn beetle species within two different climatic zones (tropical and subtropical habitats) of Yunnan province, Southwest China.

**
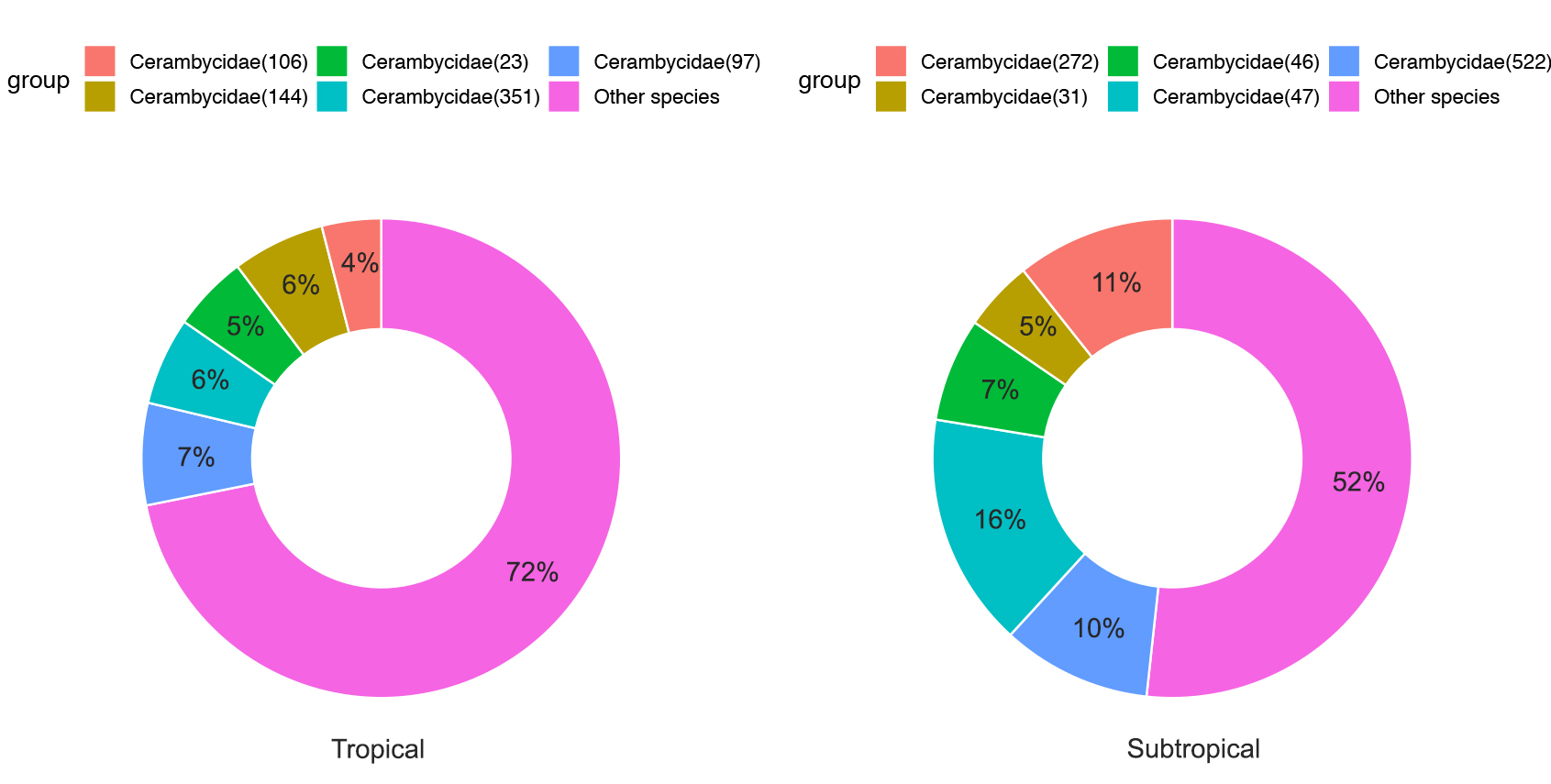
**

**Table S1.** Results of redundancy analysis testing the influence of plant species composition and plant phylogeny on wood-boring longhorn beetle species composition in tropical and subtropical areas in Yunnan province, SW China. Pla, plant species; PlaPhy, plant phylogeny; Spa, spatial distance. *F* statistics to test the significance of variables were calculated using the iterative ‘anova.cca’ in ‘vegan’ using R. Significance. codes: 0 ‘***’ 0.001 ‘**’ 0.01 ‘*’ 0.05 ‘.’ 0.1 ‘ ’ 1

|  |  | | Number of plant species/phylogeny PC axes included |  | *P* |
| --- | --- | --- | --- | --- | --- |
| Tropics | | Pla | 5/15 (2,3,4,7,15) |  | 0.02 (*) |
|  | | Spa | 3/4 (1,3) |  | 0.03 (*) |
|  | | PlaPhy | 6/15 (1,2,4,5,6,7) |  | 0.04 (*) |
| Subtropics | | Pla  Spa | 3/15(2,4,15)  2/3 (1,3) |  | 0.045 (*)  0.02 (*) |
|  | | PlaPhy | 1/15 (2) |  | 0.005 (**) |
